# Photostability of 2D Organic-Inorganic Hybrid Perovskites

**DOI:** 10.3390/ma7064789

**Published:** 2014-06-20

**Authors:** Yi Wei, Pierre Audebert, Laurent Galmiche, Jean-Sébastien Lauret, Emmanuelle Deleporte

**Affiliations:** 1 Laboratoire Aimé Cotton, Ecole Normale Supérieure de Cachan, CNRS, Université Paris Sud, Bâtiment 505, 91404 Orsay, France; E-Mails: ywei@dlut.edu.cn (Y.W.); jean-sebastien.lauret@lac.u-psud.fr (J.-S.L.); 2 School of Physics & Optoelectronic Engineering, Dalian University of Technology, Dalian 116024, China; 3 Laboratoire de Photophysique et Photochimie Supramoléculaires et Macromoléculaires de l’Ecole Normale Supérieure de Cachan, 61 avenue du Président Wilson, 94 235 Cachan, France; E-Mails: pierre.audebert@ppsm.ens-cachan.fr (P.A.); laurent.galmiche@ppsm.ens-cachan.fr (L.G.)

**Keywords:** organic-inorganic hybrid perovskites, photostability

## Abstract

We analyze the behavior of a series of newly synthesized (R-NH_3_)_2_PbX_4_ perovskites and, in particular, discuss the possible reasons which cause their degradation under UV illumination. Experimental results show that the degradation process depends a lot on their molecular components: not only the inorganic part, but also the chemical structure of the organic moieties play an important role in bleaching and photo-chemical reaction processes which tend to destroy perovskites luminescent framework. In addition, we find the spatial arrangement in crystal also influences the photostability course. Following these trends, we propose a plausible mechanism for the photodegradation of the films, and also introduced options for optimized stability.

## 1. Introduction

During the last 30 years, 2D organic-inorganic hybrid semiconductors have been paid substantial attention owning to their favorable properties and great application potential in optodevices [1,2,3,4,5]. These self-assembled compounds form well ordered multi-layered structures crystals spontaneously. This ability enables to obtain films samples simply by spin-coating which is much more convenient than the traditional deposition technique for inorganic semiconductor such as Molecular Beam Epitaxy (MBE), MetalOrganic Chemical Deposition (MOCVD) and Plasma Enhanced Chemical Vapor Deposition (PECVD). 

These organic-inorganic crystals have a usual configuration as (R-NH_3_)_2_MX_4_, where R is an organic group, M is a divalent metal in the oxidized state (such as Cu^2+^, Mn^2+^, Sn^2+^, Fe^2+^, Pb^2+^) and X is an halogen (in the halogenide form Cl^−^, Br^−^ or I^−^). The organic ammonium groups form a layer with a low dielectric constant about 2.4 while the metal halide layers possess a high constant about 6.1 [6]. These two kinds of layers function as barriers and wells alternating with each other. In low dimensional systems, stability of excitons in quantum wells is greatly enhanced due to the confined effect and the coulomb interaction. The exciton binding energy of the typical 2D organic-inorganic perovskites is up to 300 meV and their self-assembled films exhibit bright photoluminescence at room temperature [6]. Another interesting feature of perovskites is that they combine the virtues of organic flexibility, inorganic mobility and robustness in a single molecule scale. It permits us to tune their optical and electrical properties by changing either the organic or the inorganic component.

Recently, the perovskites have been used in many research fields such as surface plasmon, Light Emitting Diodes (LEDs), solar cells as well as microcavities [7,8,9,10,11,12,13,14]. Although perovskites display a much higher stability than organic semiconductors, it is still quite important to observe and optimize the long time stability of perovskite layers to support the applied requirements. In order to match these requirements, a finer study of the perovskites degradation is therefore necessary, along with an insight into the degradation mechanisms. There are two general chemical processes that can be invoked for explaining the photodegradation of perovskites: 

(a) Generation of an X^●^ radical and its becoming.

The PbX_4_^2−^ octahedra form a well structured inorganic matrix which effectively absorbs (and reemits) light. Actually, it has been long known that the optical absorption energies depend mainly on the electronegativity of halogenide involved. The absorption wavelength respectively belongs to the UV range for the chloride types, the violet range for the bromide and the green range for the iodide, the organic part playing only a minor role on the wavelength tuning. Identifying coarsely the absorption energies to the energies of an ionic bond Pb^2+^-X^−^ in an octahedral configuration [15,16], it seems very likely that the excited state corresponds to an electron transfer between the lead and one halogenide and therefore takes the transient Pb**^●+^**-X**^●^** configuration. Consequently the photostability of the perovskites would be linked to the becoming of the photogenerated X**^●^** radical in its local environment, especially if it can generate dihalogens (I_2_, Br_2_, and Cl_2_). In Kitazawa’s work, they indeed reported that the oxidation induced halogen elimination is one possible reason for degradation [17]. On the other hand, the X**^●^** radical itself may react with the organic spacers in the case they are aromatic or present a reactive hydrogen.

(b) Photodeprotonation of the ammonium ion, followed by amine oxidative degradation.

The ammoniums ions are located close to the lead halide plans. Although no literature evidence did already consider this, it is quite possible that photoexcitation enhances the basicity of the octahedral top halide ions, which can in consequence induce deprotonation of the ammonium, leaving amines now very vulnerable to photooxidation.

All these mechanisms can be investigated through the choice of appropriate methods: In this article we describe an extensive analysis of the perovskites photodegradation; we have especially set up a series of experiments allowing us to distinguish between the possible mechanisms described above, and especially discriminating between the mechanisms, while excluding other ones, on the basis of both the perovskite composition and degradation kinetics.

## 2. Experimental Section

### 2.1. Synthesis of the Perovskites

The ammonium salt precursor of the perovskite was prepared by bubbling a flow of dry HI or HBr gas into a dry ethereal solution of the amine. The following amines and the aqueous acids (99%) were purchased (Aldrich Chemicals, Sigma-Aldrich Chemie S.a.r.l. L’Isle d’Abeau Chesnes, France) and used without purification. The names below refer to the corresponding perovskites in the paper (the last letter, I, standing for iodide and B for bromide). Solutions of perovskites were prepared by mixing stoichiometric amounts of the amine salt with the corresponding lead halide.

### 2.2. Layers Preparation

Each ammonium salt was mixed with PbI_2_ or PbBr_2_ in a stoichiometric ratio of 2:1, and then dissolved in dimethylformamide (DMF, Sigma-Aldrich Chemie S.a.r.l. L’Isle d’Abeau Chesnes, France) at a 10% or 1% mass ratio (according to what specified). The solution was dispersed on a glass substrate by spin-coating at 1500 rpm for 30 s, and the self-assembled 2D perovskite layers were formed, and then annealed for 1 h at 60 °C. With these parameters, the thickness of a PEPI organic–inorganic perovskite layer was measured by Dektak profilometer to be about 50 nm (more details on the layers preparation can be found in [12,13,14]).

### 2.3. Optical Measurements

**Figure 1:**
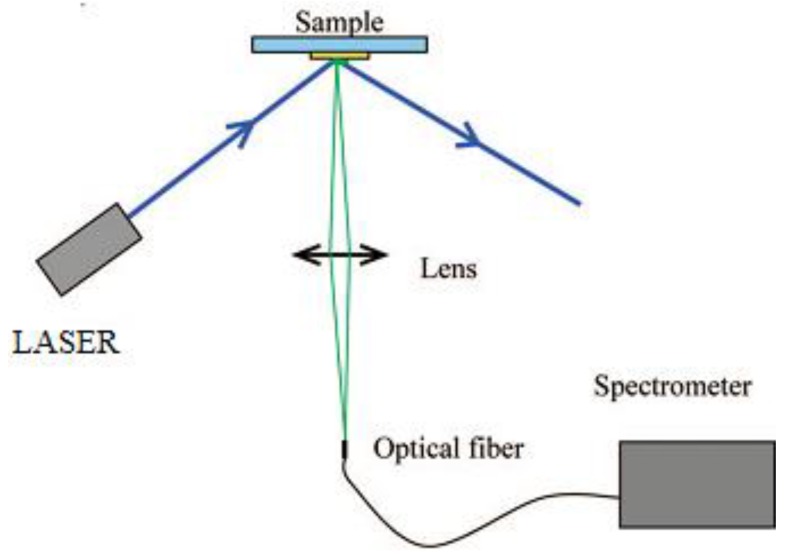
Photoluminescence recording set-up.

The photoluminescence (PL) spectra of the particles were obtained with the apparatus described in Figure 1 below, and the excitation was made through a laser diode at 405 nm (for iodide based perovskites) or a He Cd laser at 325 nm (for the bromide based perovskite).

## 3. Results and Discussion

### 3.1. Degradation through HX Photoelimination

As stated in the introduction, a possible mechanism for photodegradation could be photo-assisted HX (especially HI) elimination from the perovskites. To check it, we prepared some (C_6_H_5_C_2_H_4_NH_3_)_2_PbI_4_ (named PEPI hereafter) deuterated on the nitrogen atom (“heavy PEPI”) where all hydrogen atoms on the terminal -NH_3_^+^ part of the ammonium group had been exchanged for deuterium (-ND_3_^+^). In doing this, we would expect a strong difference in the behavior between PEPI and “heavy PEPI” if proton abstraction occurred at the key degradation step. For the preparation, we simply dissolve the C_6_H_5_C_2_H_4_NH_3_I (the ammonium precursor of PEPI, named PEI hereafter, Sigma-Aldrich Chemie S.a.r.l. L’Isle d’Abeau Chesnes, France) ammonium salt in 99.98% heavy water D_2_O; the protons can exchange fast, and, since the excess of heavy water protons is huge (>1000) it can be considered that fully deuteriated PEI (“heavy” PEI) is formed in solution. The PEI in D_2_O solution is then kept in a dry box in the presence of an abundant quantity of phosphorus anhydride. A week later, all the water (including the unreacted heavy water) has been sucked out and the dry crystals of heavy PEI have formed at the bottom of the recipient (for characterizations see Electronic Supplementary Information (ESI)).

**Figure 2:**
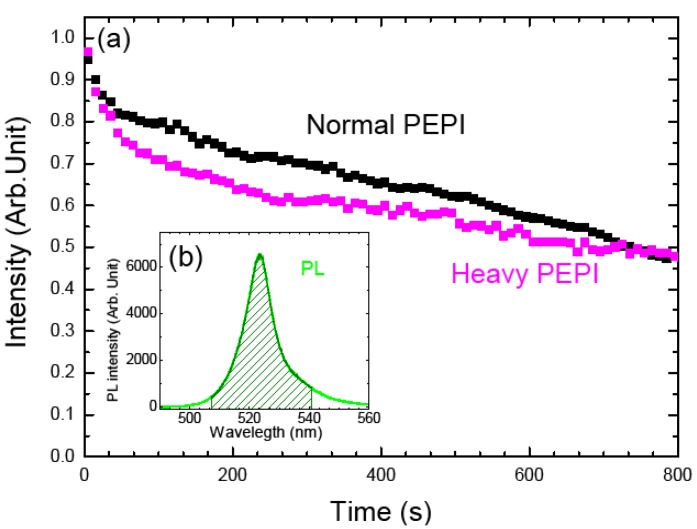
(**a**) Evolution of photoluminescence (PL) intensity as a function of illumination time for normal PEPI (black scatters) and “heavy” PEPI (magenta scatters). The samples are excited with the 325 nm line of a HeCd laser at 7 mW (corresponding to a fluence 0.175 W·cm^−2^) in air. The PL intensities are normalized to 1 at *t* = 0 s. The insert graph (**b**) is the PL spectrum of a 50 nm thick normal PEPI film measured at room temperature. The excitation source for PL measurement is the 325 nm line of a HeCd laser, with an incident power of 0.6 mW (corresponding to a fluence 0.015 W·cm^−2^).

Then the standard PEPI perovskite and the “heavy PEPI perovskite” are prepared as usual: these organic ammonium salts are dissolved with their corresponding lead halide powder in stoichiometry of 2:1 in DMF (Dimethylformamide) solvent, and then films samples are deposited by spin-coating on a quartz substrate at a speed of 2000 rpm for 30 s, and are annealed at 95 °C during 1 min. Thin films of heavy PEPI and standard PEPI were submitted to photobleaching measurements, following the luminescence intensity decrease with time (Figure 2a, the luminescence intensity is here the integral of the PL peak as seen in Figure 2b (hatched zone). Although -NH_3_^+^ and -ND_3_^+^ are greatly different in hydrogen bond force and acid-base properties, the change does not affect the perovskites’ stability. Therefore, we can conclude that the breakage of hydrogen bond H-N does not take place at the key step of the degradation.

### 3.2. Oxidation and Elimination of Halogen Species in Inorganic Parts

**Figure 3:**
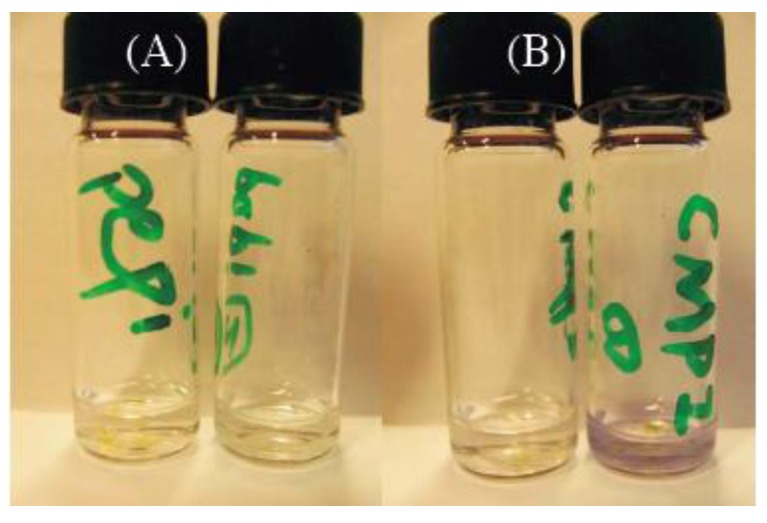
Results of the photodegradation of two perovskites (**A**) PEPI (containing a phenyl ring) and (**B**) a lead perovskite CMPI (alkyl based), both immersed in cyclohexane. On the left stand the vials before irradiation, and on the right after irradiation. The colour comes from a starch-based iodine revealing substance, and shows the relative release of traces of molecular iodine. The CMPI alkylammonium based perovskite photodegradation releases a quite discernible and rather largeramount of iodine than the PEPI phenylammonium based perovskite.

If halide radicals are produced during the photodegradation, they can react through two main paths. First, if the organic moiety is unreactive, they can couple to form molecules of halogen (iodine, bromine or chlorine resp.), which are either gaseous of low boiling and can quickly get out of the film. On the other hand, in the case of a more or less reactive organic part, they can substitute, or add to it, leading probably to an even faster degradation. While it is quite hard to put in evidence the production of trace amounts of chlorine or bromine, this is feasible in the case of iodine, because effective colorimetric revelators of iodine traces (related to starch) are now commercially available: we used Iotect indicator (No. CAS: 9005-84-9), which is starch glue mixed with urea. In fact, we checked in an experiment that long term illumination of iodide based perovskites indeed induces the elimination of iodine. The photo of Figure 3B shows the result of two parallel experiments. It shows two bottles of solution in which an amount of iodine indicator was dissolved in cyclohexane, while thin films of (C_6_H_11_CH_2_NH_3_)_2_PbI_4_ (named CMPI hereafter) are immersed in each bottle of solution. Before illumination, the two solutions are transparent and colorless. Then, the bottle containing CMPI on the right is exposed to the illumination of a 325 nm line of a HeCd laser at 27 mW (corresponding to a fluence 0.675 W·cm^−2^) during 2 h, while the other one is kept aside as a control. We see clearly that in Figure 3B the solution in the right bottle becomes slightly purple after illumination, which indicates that some iodide radicals have been eliminated from CMPI and have formed iodine I_2_ appearing in solution. However, the iodine content is quite dependant on the organic part. If we carry on the same experiment with PEPI thin film as seen in Figure 3A: we see in the right hand bottle under illumination that much less iodine appears in the solution (only a hint of darkening is discernible). It is likely that the aromatic groups are able to react with the iodide radicals (probably through a radical substitution on the aromatic ring, or on the a-methyl group), while unreactive spacers (e.g., aliphatic) on the other hand would not react and lead in fine to the generation of molecular iodine from pseudo-radical coupling in the solid.

**Figure 4:**
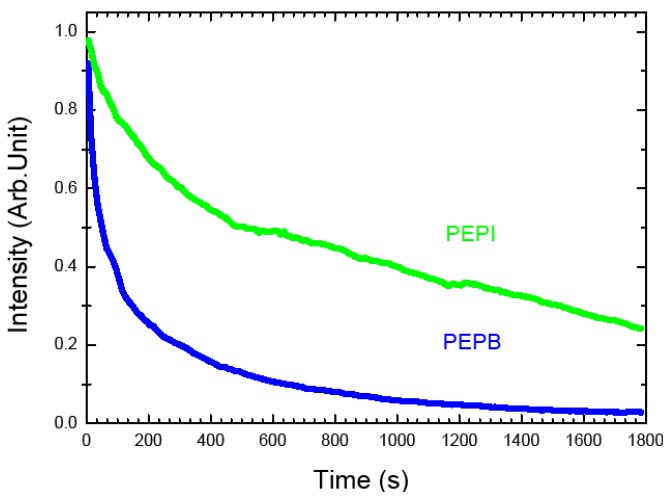
Evolution of PL intensity as a function of illumination time for PEPI, PEPB thin films.The samples are excited with the 325 nm line of a HeCd laser at 7 mW (corresponding to a fluence 0.175 W·cm^−2^) in air. The PL intensities are normalized to 1 at *t* = 0 s.

We also performed experiments showing the degradation of two different perovskites, respectively bromide and iodide based, but having the same phenylethanamine (named PE hereafter) amine part (so that for example PEPB is (C_6_H_5_C_2_H_4_NH_3_)_2_PbBr_4_). If degradation were to be related to photogenerated radical species, the bromide perovskites should be less stable because of the higher reactivity of the bromide radical. Actually this is the trend which was found throughout experiments (see Figure 4), lead bromide perovskites are less stable than lead iodide perovskites upon irradiation, while however the former group generally exhibits at the beginning a stronger PL than the latter group.

### 3.3. Photostability Dependence upon the Structure of the Organic Part of Bromide and Chloride-Based Perovskites

Considering the previous results on the iodine detection, it seemed likely that radicals are photogenerated in the degradation process. Since we expect that bromide and chloride radicals are more reactive than the iodine one, we examined the behavior of a large range of bromide and chloride based-perovskites and we especially focused on how the degradation depends on the ammonium structure. The Table 1 shows the different amines that we have used to synthesize these perovskites. Details about the syntheses of the perovskites have been reported elsewhere [18,19].

**Table 1:** Chemical structures, abbreviations, names and chemical equations for the amine precursors of the perovskites.

Chemical structure of amines (Abbr.)	Name	Chemical formula
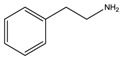 (PE)	2-Phenylethanamine	C_6_H_5_C_2_H_4_NH_2_
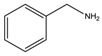 (PM)	Phenylmethanamine	C_6_H_5_CH_2_NH_2_
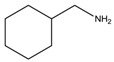 (CM)	Cyclohexylmethanamine	C_6_H_11_CH_2_NH_2_
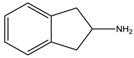 (A5)	2-Aminoindan	C_6_H_4_C_3_H_5_NH_2_
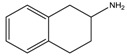 (TNY)	1,2,3,4-Tetrahydro-naphthalen-2-ylamine	C_6_H_4_C_4_H_7_NH_2_
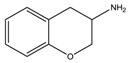 (CY)	Chroman-3-ylamine	C_6_H_4_C_3_H_5_ONH_2_
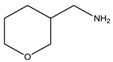 (TPMA)	C-(Tetrahydro-pyran-3-yl)-methylamine	C_5_H_9_OCH_2_NH_2_

**Figure 5:**
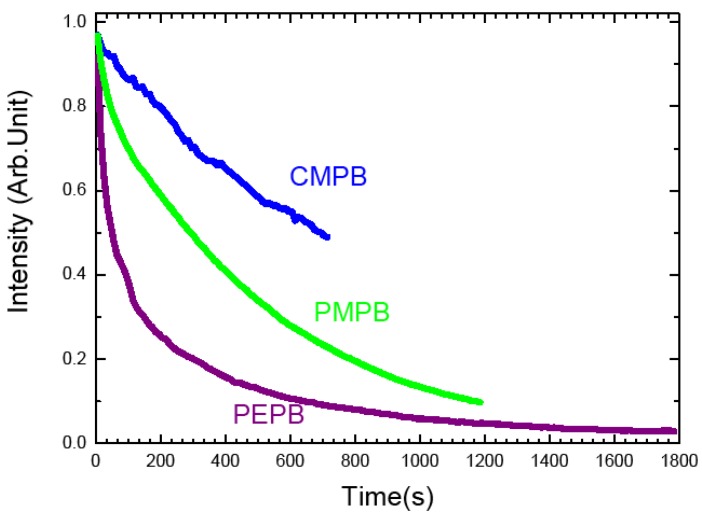
Evolution of PL intensity as a function of illumination time for PEPB PMPB and CMPB thin films. The samples are excited with the 325 nm line of a HeCd laser at 7 mW (corresponding to a fluence 0.175 W·cm^−2^) in air. The PL intensities are normalized to 1 at *t* = 0 s.

The photostabilities of the bromide based perovskites that we examined are quite different indeed, according to the type of ammoniums. It can be seen on Figure 5 that the aliphatic ammonium based CMPB is much more resistant than the phenyl-based ammoniums PMPB and PEPB, PEPB being as expected the less photostable, presumably because of its more electron-rich character.

**Figure 6:**
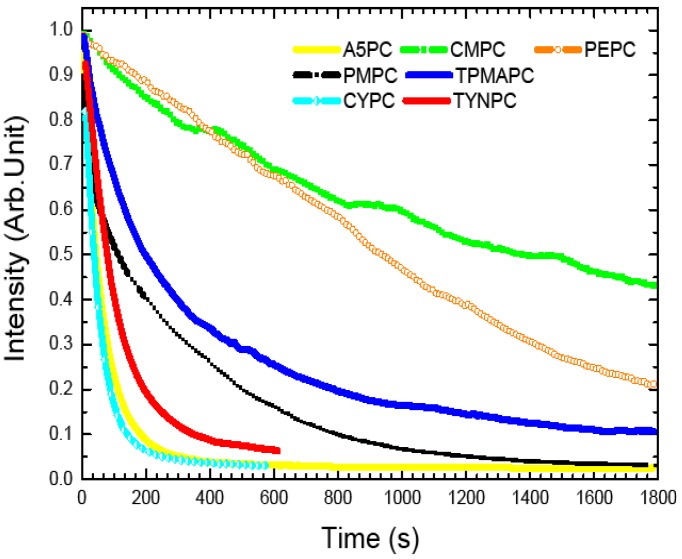
Evolution of PL intensity as a function of illumination time for PEPC, PMPC, CMPC, A5PC, TPMAPC, TNYPC and CYPC thin films in air. The excitation source is the 325 nm line of a HeCd laser, the laser power is fixed at 7 mW (corresponding to a fluence 0.175 W·cm^−2^). The PL intensities are normalized to 1 at *t* = 0 s.

In Figure 6, the photostability range of chloride based perovskites is also quite informative. Ranging from a strong to a weak resistance in this series, we find: CMPC > PEPC > TPMAPC > PMPC > A5PC/TNYPC > CYPC. It appears that the most resistant perovskites come again from the ammoniums with a saturated backbone, while the aromatic ones degrade faster, as it could be expected from the reaction with a strongly electrophilic radical. In addition, among the aromatic perovskites, we see that the most resistant are the ones where the benzene ring is the less activated (PMPC and PEPC), while the ammoniums with an electron-rich dialkylbenzene, or even richer chroman-type ring, indeed degrade very fast. This is again in perfect accordance with a degradation through an electrophilic attack of a chloride radical on the aromatic part in the ammonium.

### 3.4. Photostability of Some Resistant Fluorinated Perovskites

**Figure 7:**
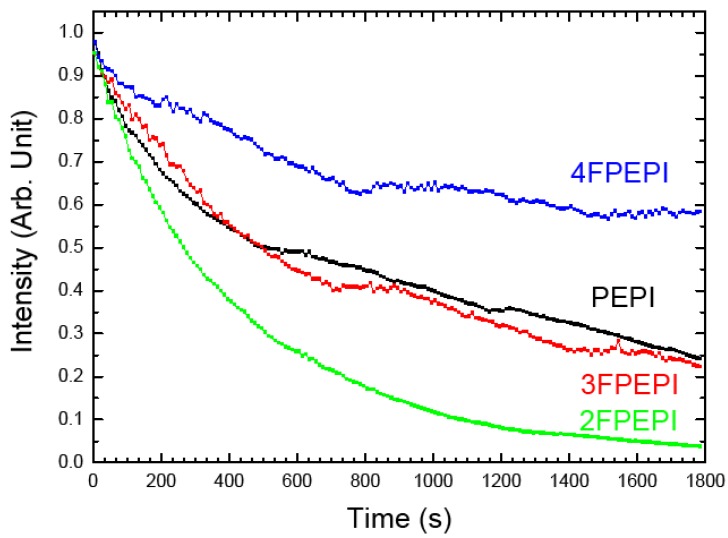
Evolution of PL intensity as a function of illumination time for fluorinated perovskites. The excitation source is the 325 nm line of a HeCd laser, the laser power is fixed to be 7 mW (corresponding to a fluence 0.175 W·cm^−2^).

In order to overcome the degradation through electrophilic attack on perovskites, we have prepared a family of fluorinated ammonium iodide based perovskites. We more particularly synthesized some fluoro-PEPI perovskites whose organic amine has a fluorine atom substituted on the three different available positions of the benzene ring, resulting in respectively the ortho-, meta- and para- fluorophenethylamine tetraiodoplumbate, respectively designed by 2FPEPI, 3FPEPI and 4FPEPI [20]. This was done because the introduction of the fluorine atom on the ring considerably decreases its reactivity to electrophilic reagents. These perovskites exhibit bright green photoluminescence around 520 nm at room temperature. Although they have a similar steric hindrance, the stability behaviors of these amines based perovskites differ under intense irradiation, as can be seen on Figure 7. We noticed that 2FPEPI and 3FPEPI are the less stable materials in this group. Interestingly, the Stokes-shift of 2FPEPI has a somewhat high value of 6.3 nm, obviously larger than the others and its PL peak shifts from lower energy to higher before and after partial photobleaching (Table 2).

**Table 2:** PL peak shifts before and after long-time illumination of UV laser and Stokes-shift comparison

Name	Shifts of PL Peak Center	(Difference)	Stokes-Shift
PEPI	523.83 nm → 523.21 nm	(0.62 nm)	4.96 nm
2FPEPI	515.34 nm → 513.32 nm	(2.02 nm)	6.3 nm
3FPEPI	517.94 nm → 516.9 nm	(1.04 nm)	5.1 nm
4FPEPI	524.96 nm → 524.13 nm	(0.83 nm)	4.05 nm

**Figure 8:**
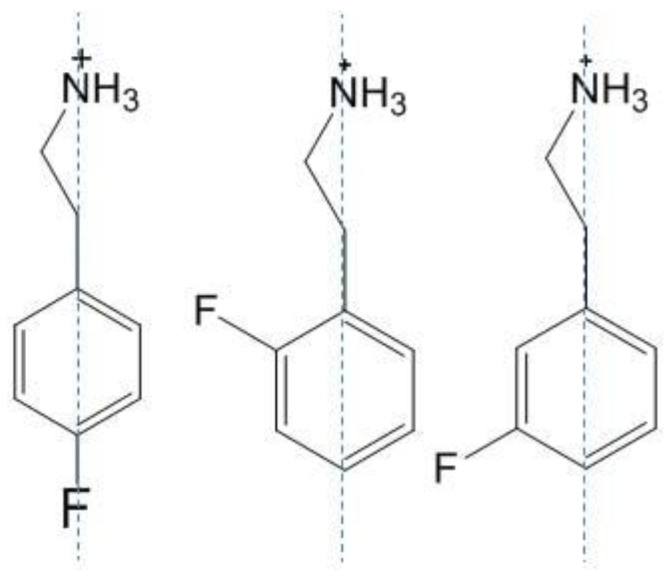
Schema showing the arrangement of the ammoniums in fluorinated perovskites.

The reason for the Stokes-shift evolution is not very clear, but it may be attributed to the occurrence of lattice defects with the photodegradation. We suppose that defaults in superlattices can be more or less important in relation with the dissymmetry of the amine configuration. As can be seen in Figure 8, 4FPEPI and PEPI have a more symmetrical structure thus degradation probably induces less disorder in the arrangement of the lattice. This is also supported in [21] by the fact that (4FC_6_H_4_C_2_H_4_NH_3_)_2_SnI_4_ is a fully ordered structure while (2FC_6_H_4_C_2_H_4_NH_3_)_2_SnI_4_ and (3FC_6_H_4_C_2_H_4_NH_3_)_2_SnI_4_ are much less ordered due to the mismatch between sheets [21].

### 3.5. Influence of Non-Composition Dependant Parameters

#### 3.5.1. Solution Concentration

**Figure 9:**
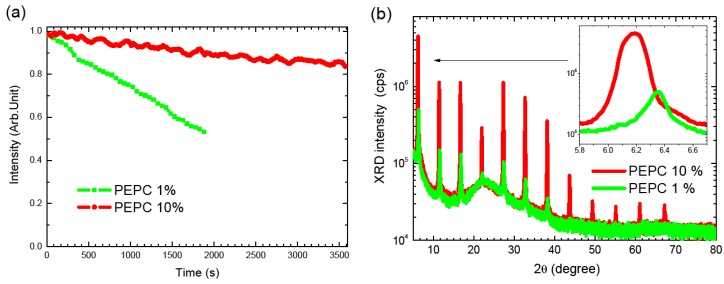
(**a**) Evaluation of PL intensity as a function of illumination time for two PEPC thin filmsprepared from 1% and 10% in dimethylformamide (DMF) solution. The laser power is 7 mW at 325 nm (corresponding to a fluence 0.175 W·cm^−2^); (**b**) XRD spectra of PEPC thin films prepared from 1% (green line) and 10% (red line) in DMF solution.

2D perovskites are layered structures containing an organic sheet of about 1 nm and an inorganic sheet of about 0.6 nm. They spread out infinitely in 2D plane, named the a-b plane. The layers stack together layer by layer along the c axis direction. It is generally admitted that there is small difference in the *d*_c_ value with the deposition conditions (this value keeps around 1.6 nm) for 2D perovskites. However, the *d*_a_, *d*_b_ values, might vary according to the deposition parameters (especially the concentration of the spin-coating solution), and may have a large effect on the stability of perovskites crystalline stacking. We observed that some perovskites films prepared from solutions of different concentrations exhibit different photostabilities. For example PEPC films deposited from 1% solution and 10% solution have very different degradation kinetics under UV illumination, as we can see on Figure 9a. The XRD spectra of the two film samples of PEPC show that both films are well oriented and crystalline with a single phase present; however, comparing the position of the diffraction peaks, we see a clear difference in the displacement comparing the two curves in Figure 9b (insert), as already noticed by Kitazawa [22]. While the real reason for this discrepancy in stability with the deposition conditions (and crystalline lattice) is not clear, it indicates nonetheless that the solution concentration is an important parameter to consider.

#### 3.5.2. Influence of Temperature

**Figure 10:**
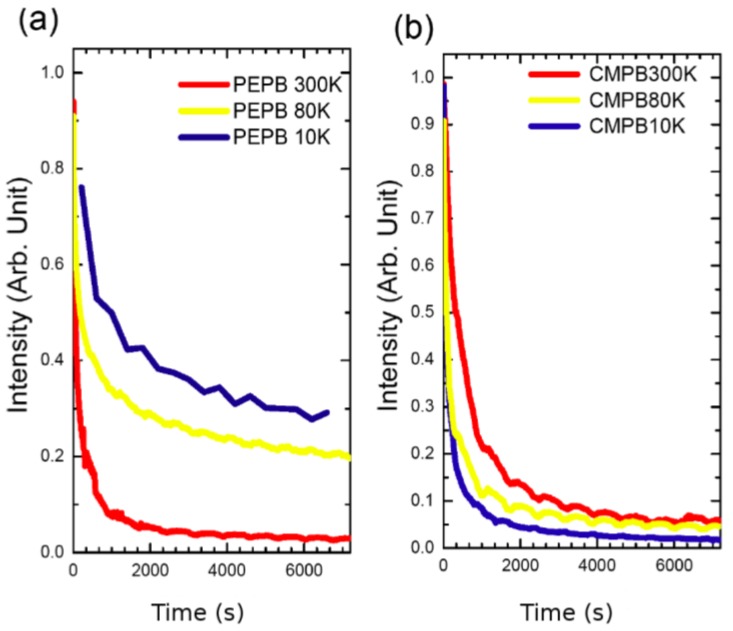
Evolution of PL intensity as a function of illumination time at different temperatures. The laser power is 7 mW (corresponding to a fluence 0.175 W·cm^−2^) at 325 nm. (**a**) PEPB and (**b**) CMPB in helium environment. The influence of temperature is atypical for CMPB.

In addition to other parameters, we have observed that the photostability of a given perovskite, especially in the case of bromide based ones, is temperature dependent. Figure 10a shows the evolution of PL intensity as a function of time for a PEPB thin film at 10 K, 80 K and 300 K under 10 mW illumination of a HeCd laser (corresponding to a fluence 0.25 W·cm^−2^) under an helium gas atmosphere. We see that the PEPB film is the most stable at 10 K and the least stable at 300 K as it could be expected. However, this expected evolution is not always observed. For example, CMPB has surprisingly the best stability at 300 K, as can be seen on Figure 10b. This phenomenon, although surprising, might be associated with the mixed kinetics of evaporation and chemical attack of the bromide radicals, the slower chemical attack on aliphatic ammoniums leading to an apparent inversion in the expected temperature dependence.

Also in the case of an attack by an electrophilic radical, in the case of a purely aliphatic ammonium, where the reactivity is low and has slow kinetics, the bromation of the aliphatic ammonium can be in competition with dimerization trapping of the radicals, which leads to faster departure of bromine gas with the increasing temperature. On the other hand, in the case of PEPB, the main degradation path is a radicalar attack on the alkylbenzene ring (radical coupling is always much slower and is not observed), and degradation normally occurs more quickly with increasing temperature.

## 4. Conclusions

We have performed a relatively complete study on the photostability issue of some (R-NH_3_)_2_PbX_4_ perovskites. Based on photobleaching experimental results, we have been able to exclude proton exchange photoinduced mechanisms, while on the other hand we have shown that there is a strong presumption of degradation through electrophilic attack of photogenerated halide radicals; the presence of iodine has also been evidenced in the case of aliphatic ammoniums. To reduce the UV induced oxidation to perovskites molecules, we have prepared some fluorinated perovskites: 4FPEPI shows a best photostability than PEPI. Another method that can be done is to protect the perovskites film with another optical transparent material such as PMMA polymer (PolyMethyl-MetAcrylate) [23,24,25]. We also fabricated films in which perovskites are protected by PMMA polymer layer [14] and it has been proved that these layers exhibit a higher stability under irradiation. Our research certainly provides a wider prospect for perovskites applications in optical devices and scientific research.
